# Incidence, aetiology and clinical features of eosinophilic pleural effusion: a retrospective study

**DOI:** 10.1186/s12890-021-01767-1

**Published:** 2021-12-06

**Authors:** Minfang Li, Yunxiang Zeng, Yaqing Li, Dan Jia, Sheng Chen, Jinlin Wang

**Affiliations:** 1 Department of Respiratory Medicine, Shenzhen Traditional Chinese Medicine Hospital, Shenzhen, 518033 Guangdong Province China; 2 The Second School of Clinical Medical Sciences, Guangzhou University of Chinese Medicine, Guangzhou, 510120 Guangdong Province China; 3 Department of Respiratory Disease, The State Key Laboratory of Respiratory Disease, China Clinical Research Centre for Respiratory Disease, Guangzhou Institute of Respiratory Health, First Affiliated Hospital of Guangzhou Medical University, 151 Yanjiang Road, Guangzhou, 510120 Guangdong Province China

**Keywords:** Eosinophilic pleural effusion, Eosinophils, Pleural effusion, Pleural fluid, Pleural fluid eosinophilia

## Abstract

**Background:**

Eosinophilic pleural effusion (EPE) is a distinct entity among pleural effusions, but its diagnostic and prognostic significance is still controversial. This study aimed to evaluate the incidence and aetiological distribution of EPE in our institution and to assess the relationship between EPE and malignancy and other underlying diseases and the relevance of the percentage of eosinophils and other laboratory parameters.

**Methods:**

A retrospective study was conducted by reviewing the medical records of 252 patients with PE from September 2017 to January 2021.

**Results:**

EPE was found in 34 (13.49%) out of 252 patients. There were 20 (58.82%) males and 14 (41.18%) females in the EPE group. The mean percentage of eosinophils in EPE (21.7%, range (10.0–67.5%)) was significantly higher than the percentage of eosinophils in peripheral blood (5.65%, range (0–34.60%); *p* < 0.05). The most common cause of EPE was malignant disease (52.94%), followed by idiopathy (14.71%), parasites (8.82%), pneumonia (8.82%) and others (14.71%). Comparative analysis of patients with malignant *versus* nonmalignant EPE showed that patients with malignant EPE were significantly older, and had a lower white blood cell (WBC) count in the pleural fluid (1.8 vs 4.7 cells × 10^9^/L, *p* < 0.05). However, the percentage of eosinophils in PE was not significantly different between malignant EPE and nonmalignant EPE (*p* = 0.66). There was no correlation between the percentage of eosinophils in PE and peripheral blood (r = 0.29; *p* = 0.09).

**Conclusions:**

Malignant disease ranks as the leading cause of EPE. The presence of EPE should not be considered as a predictive factor of benign conditions. Pleural parasitic infestation (PPI) should be emphasized in areas with a high incidence of parasitic disease.

## Background

Eosinophilic pleural effusion (EPE) was first described by Harmsen in 1894 [1]. Since then, it has been of interest to clinicians. EPE, defined as a pleural effusion (PE) in which eosinophils comprise ≥ 10% of white blood cells (WBCs) [2], accounts for 5–16% of exudative pleural effusions [2, 3].

The diagnostic value and prognostic significance of EPE are still a matter of debate, as it can be a manifestation of a great variety of diseases including infections (bacteria, fungi, mycobacteria, parasites), malignancies, autoimmune diseases, drug reactions, pulmonary embolism, chest trauma, asbestos exposure and many others [2–5]. Early studies reported that air/blood was the most common cause of EPE (29%), and it was once believed that the finding of pleural fluid eosinophilia in an exudative effusion considerably reduced the probability of malignancy and conversely increased the likelihood of an underlying benign disorder [2–5]. However, current studies, performed in the last 2 decades, have confirmed that malignancy accounts for 22.7- 40.1% of EPEs and is the most common aetiology of EPEs [4–9]. This disparity could probably be explained by different study populations, and the various disease spectra of EPEs were a reflection of the populations studied [5].

Due to the rarity of EPE, our knowledge of this phenomenon is based on small series and case reports until now. We are aware of no related report in China. We performed an analysis of a series of samples of PE in our institution. This study aimed to investigate the incidence, aetiology and epidemic characteristics of EPE in our institution.

## Methods

### Study design and setting

A retrospective study of patients with PE was performed at a dedicated respiratory centre (State Key Laboratory of Respiratory Disease and China Clinical Research Centre of Respiratory Disease, Guangzhou Institute of Respiratory Disease, Guangzhou) between September 2017 and January 2021. The study design and protocol were approved by the Ethics Committee of the First Affiliated Hospital of Guangzhou Medical University, and the study was conducted in accordance with the 1964 Declaration of Helsinki and its later amendments. Approval for a waiver of informed consent for the study was obtained from the Institutional Review Board of Guangzhou Medical University.

### Patients

The available data for a total of 252 patients with PE were reviewed retrospectively.

The inclusion criteria for patients with EPE were pleural fluid containing ≥ 10% of eosinophils.

The inclusion criteria for patients with tuberculous pleural effusion (TPE) were as follows: (1) chronic granulomatous inflammation in pleural tissue; (2) a clinical response to anti-tuberculosis treatment; and (3) no pleural effusion or only a small amount observed in chest ultrasound examinations over a 12-month follow up period.

The criteria of MPE (malignant pleural effusion) were: (1) a positive pleural fluid cytology and/or positive histology of pleural biopsy (proven malignant effusion); or (2) a known malignant disease, after the exclusion of alternative causes of PE (probable malignant effusion) [7].

The criteria for inclusion of patients with PPE (parapneumonic effusion) were as follows: (1) exudative effusions associated with bacterial pneumonia, lung abscesses, or bronchiectasis; (2) absence of *Mycobacterium tuberculosis (*MTB) in pleural fluid obtained from serial thoracentesis procedures; (3) pathological manifestations of inflammatory pleuritis, pleural fibrosis and plaques, or chronic empyema, without evidence of MTB; and (4) remission and recovery for at least 3 months at follow-up visits after antibiotic treatment.

The inclusion criteria for enrolment of patients with PPI (pleural parasitic infestation): (1) parasite exposure; (2) immunoserologic test result for a parasite-specific antibody, and/or on the detection of characteristic parasite eggs (in the pleural effusion, sputum, bronchial washing fluid, lung biopsy specimens or stool); (3) patients with presumptive diagnosis of PPIs who were responsive to antiparasitic treatment and were followed up for up to 15 months.

CTDs (Connective tissue diseases) were diagnosed in patients with a known specific CTD after the exclusion of other causes of PE.

HRPE (heart related pleural effusion) was identified in patients with definite heart disease followed by exclusion of other causes of PE.

The aetiology of PE was established based on the medical history, physical examination, imaging studies, laboratory findings and pleural fluid and pleural biopsy examination results. Almost all cases of idiopathic eosinophilic pleural effusions (IEPE) had sufficient follow-up periods to exclude malignancy or tuberculosis (more than 1 year).

### Statistical analysis

Continuous variables are presented as the median and range or the mean and standard deviation, and qualitative variables are presented as the number and percentage. Intergroup differences were analysed statistically using SPSS® 19.0 (SPSS Inc., Chicago, IL, USA). The Mann–Whitney test or Chi-square test was used for comparisons of the test results. Significance for statistical analyses was set at *p* < 0.05.

## Results

### Patient characteristics

EPE was found in 34 (13.49%) out of 252 patients. There were 20 (58.82%) males and 14 (41.18%) females in the EPE group, and the male to female ratio of nearly 1.43:1 was lower than that for all patients with PE seen during the study period (1.8:1). The mean age of patients in the EPE group was 56.41 ± 15.3 years (range 25–88 yreas). Their clinical characteristics are shown in Table 1. The symptoms presented by the patients included dry cough, shortness of breath, fever, chest pain, and sputum. The duration of complaints ranged from 2 days to more than 2 years. Twenty-seven (79.41%) patients had unilateral PE: 16 (59.26%) left-sided and 11 (40.74%) right-sided. Bilateral PEs were found in 5 (14.71%) patients, and polyserositis occurred in 2 (5.88%) patients. Thirty-three (97.06%) EPEs were identified as exudates, and one (2.94%) was identified as a transudate.

**Table 1:** Demographic characteristics of the 34 patients with EPE

No	Age (y)	Sex	Peripheral blood					Pleural effusion						Diagnosis
			WBC (× 10^9^/L)	LYM (× 10^9^/L)	EO (× 10^9^/L)	WBC (× 10^9^/L)	EO (%)	RBC (× 10^6^/L)	LDH (U/L)	ADA (U/L)	GLU (mmol/L)	Protein (g/L)	CEA (ng/mL)	
1	48	M	4.7	0.3	0.6	4.4	13.5	1466	128	6.7	3.02	43.2	4.80	PPI
2	29	M	12.7	1.6	0.3	5.8	30.2	1703	587	19.1	3.19	43.1	1.74	PPI
3	63	F	16.7	1.8	5.8	8.0	22.5	449	667	10.8	5.95	47.4	0.30	PPI
4	48	M	11.3	1.2	0.6	4.8	19.0	2200	189	2.8	7.79	39.8	1.67	IPE
5	49	M	4.6	1.4	0.4	3.8	15.2	5277	201	24.0	5.15	35.6	4.31	IPE
6	45	M	8.6	0.7	0.4	4.7	21.2	3251	234	20.0	4.84	43.2	3.34	IPE
7	52	F	8.7	1.5	0.3	7.4	18.9	549	321	17.0	4.97	34.2	2.67	IPE
8	49	F	10.4	1.1	0.8	5.5	12.4	1583	214	19.9	4.40	32.8	3.40	IPE
9	35	F	5.7	1.4	0.3	6.6	46.5	5262	421	20.4	5.61	49.6	0.95	PPE
10	25	M	9.7	2.4	0.7	12.9	67.5	1232	365	6.5	5.45	53.4	1.54	PPE
11	67	F	14.6	0.5	0	4.1	19.2	5637	145	1.7	8.02	33.9	2.15	PPE
12	46	M	8.1	0.9	0.1	2.6	12.8	4788	558	44.7	5.68	53.4	1.18	TPE
13	83	F	6.5	1.5	0	0.4	15.7	4581	130	2.1	6.90	27.5	1.67	HR-PE
14	33	F	7.6	0.6	0.1	0.8	15.3	186	116	0.1	5.48	28.8	1.32	CTD-PE
15	66	M	5.2	1.2	0	0.3	25.5	2,986,738	90	1.8	7.45	37.5	1.53	Haemothorax
16	70	M	5.2	0.6	0	2.4	11.6	20,185	111	3.8	6.37	32.4	2.08	Pulmonary embolism
17	70	M	4.9	0.9	0.1	3.2	23.5	2339	1402	13.2	6.11	39.5	148.20	MPE
18	57	M	9.9	2.3	1	1.1	55.0	15,319	712	10.3	6.66	48.2	162.20	MPE
19	74	M	7.7	1.2	1.5	0.3	11.0	192	326	3.1	5.99	35.2	49.04	MPE
20	45	M	6.8	1.1	0.3	1.3	11.6	7422	352	7.2	6.34	53.7	81.15	MPE
21	44	F	6.8	1.2	0.2	0.1	12.5	2573	403	4.2	5.80	60.7	2087.0	MPE
22	88	M	8.6	2.5	0.4	0.9	12.0	4951	563	3.3	4.52	49.3	384.50	MPE
23	47	F	4.9	1.1	0.2	1.5	10.5	13,366	172	3.1	7.52	40.6	289.70	MPE
24	58	M	14.8	1.1	0.1	0.4	23.5	5052	310	7.8	5.57	44.3	339.40	MPE
25	81	M	21.4	1.9	0.1	1.0	13.5	2859	182	7.6	6.04	182.9	2.60	MPE
26	59	F	5.1	1.2	0.2	0.2	14.5	25,654	424	4.2	5.19	40.5	0.94	MPE
27	61	M	6.2	0.9	0	0.6	11.5	5787	215	7.0	7.24	53.9	17.39	MPE
28	55	F	8.8	2.2	0.2	0.2	10.0	7097	217	3.0	4.44	40.2	1.27	MPE
29	67	F	9.5	1.5	0.3	4.5	11.0	320,000	327	4.2	8.34	52.0	10.97	MPE
30	76	M	6.4	1.0	0.1	1.1	10.5	3000	232	4.0	8.01	42.9	4.07	MPE
31	56	F	3.2	0.6	0.5	2.2	31.0	1752	265	8.1	7.83	51.8	1.89	MPE
32	44	M	11.2	1.5	0.6	0.4	22.5	12,315	209	4.0	9.77	49.7	0.46	MPE
33	57	F	9.0	1.8	0.8	7.7	63.0	216,195	1126	29.3	4.32	62.3	0.91	MPE
34	71	M	8.11	0.9	0.5	6.5	24.0	91,620	645	27.6	6.48	49.4	0.99	MPE

Abbreviations: *EPE* eosinophilic pleural effusion, *WBC* white blood cell, *LYM* lymphocyte, *EO* eosinophil, *RBC* red blood cell, *LDH* lactate dehydrogenase, *ADA* adenosine deaminase, *GLU* glucose, *CEA* carcinoembryonic antigen, *PPI* pleural parasitic infestation, *IPE* idiopathic pleural effusion, *PPE* parapneumonic effusion, *TPE* tuberculous pleural effusion, *HR-PE* heart related pleural effusion, *CTD-PE* Connective tissue diseases- pleural effusion, *MPE* malignant pleural effusion

### Laboratory findings

**Fig. 1:**
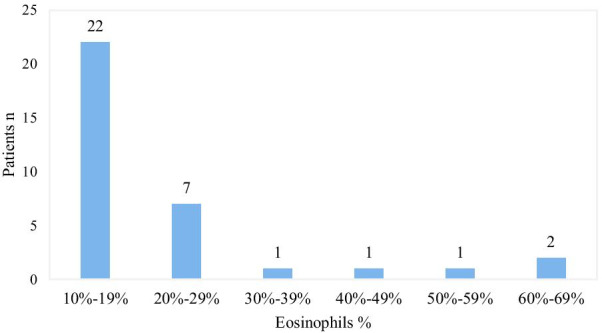
Distribution of eosinophil percentage in 34 patients with EPE

The mean percentage of eosinophils in EPE (21.7%, range (10.0–67.5%)) was significantly higher than the percentage of eosinophils in peripheral blood (5.65%, range (0–34.60%); *p* < 0.05). The mean of eosinophil count in the pleural fluid (942.4 (11.8–8682.5) cells × 10^6^/L) was also higher than that in peripheral blood (514.7 (0–5778.0) cells × 10^6^/L, *p* < 0.05). The distribution of eosinophil percentage in patients with EPE is shown in Fig. 1. Patients with less than 20% eosinophils accounted for 64.7% of patients with EPE. In 14.7% of patients with EPE, the percentage of eosinophils was greater than 30%. When eosinophilic and noneosinophilic effusions were compared, there were significant differences in the number of eosinophils in peripheral blood, the amount of WBCs and the level of adenosine deaminase (ADA) in effusions (Table 2).

**Table 2:** Comparison of clinical findings of patients with EPE and n-EPE

	Total (n = 252)	EPE (n = 34)	n-EPE (n = 218)	*p*-Value
*Age (y)*	58 (19–98)	56.4 (25–88)	58.2 (19–98)	0.56
*Sex*				0.48
Male	162 (64.3%)	20 (58.8%)	142 (65.1%)	
Female	90 (35.7%)	14 (41.2%)	76 (34.9%)	
*Peripheral blood*				
WBC (× 10^9^/L)	8.2 (3.0–42.4)	8.6 (3.2–21.4)	9.3 (3.0–42.4)	0.42
LYM (× 10^9^/L)	1.4 (0.2–13.5)	1.3 (0.3–2.5)	1.3 (0.2–13.5)	0.93
EO (× 10^6^/L)	253.7 (0–6200.0)	514.7 (0–5778.0)	168.3 (0–6200.0)	0.001
*Pleural effusion*				
LDH (U/L)	943 (25- 44,144)	369 (90–1402)	1098 (25- 44,144.0)	0.30
GLU (mmol/L)	5.80 (0.12–50.21)	6.07 (3.02–9.77)	5.70 (0.12–50.21)	0.64
Protein (g/L)	77.1 (5.2–182.9)	48.03 (27.5–182.9)	47.8 (5.2–57.3)	0.66
ADA (U/L)	19.5 (0.1–196.7)	10.4 (0.1- 44.7)	22.4 (0.1–196.7)	0.007
CEA (ng/mL)	715.60 (0.2–69,984.0)	106.39 (0.30–2087.0)	770.80 (0.2–69,984.0)	0.74
WBC (× 10^9^/L)	1.5 (0–12.9)	3.2 (0.1–12.9)	1.2 (0–8.6)	0.001
EO (%)	5.0 (0–67.5)	21.7 (10.0–67.5)	1.3 (0–8.0)	< 0.01
RBC (× 10^9^/L)	44.6 (0.08–990,000)	111.2 (0.2–2986.7)	30,607.0 (0.7–990,000)	0.36

Data are presented as the mean (range) or n (%)

*EPE* eosinophilic pleural effusion, *WBC* white blood cell, *LYM* lymphocyte, *EO* eosinophil, *RBC* red blood cell, *LDH* lactate dehydrogenase, *ADA* adenosine deaminase, *GLU* glucose, *CEA* carcinoembryonic antigen

### Aetiology of EPE

The aetiological distribution of EPEs is exhibited in Table1. The most common cause of EPE was malignant disease (52.94%) followed by IEPE (14.71%), PPI (8.82%), PPE (8.82%) and others (14.71%). Lung adenocarcinoma accounted for more than half of all MEPE (14, 77.78%). The other malignancies were pancreatic cancer, hepatic epithelioid haemangioendothelioma, lung squamous cell carcinoma, and colorectal cancer. In 14.71% of patients diagnosed with IEPE, the origin of EPE remained unknown after extensive evaluation, including pleural biopsy. The quantity of their pleural effusion was small and did not relapse after the first thoracentesis. None of these patients had an exposure history to asbestos or drugs known to lead to EPE.

Among the 5 patients with PPIs, 2 had a history of raw fish ingestion or raw freshwater crab ingestion. The other 3 patients did not report an exposure history. IgG antibodies against *Paragonimus westermani* were found in the serum of 3 patients. *Echinococcus granulosus* and *Taenia solium* specific antibodies were detected in the blood samples of 2 other individuals. The diagnosis of PPI was suspected and the patients were treated with antiparasitic agents. All of them recovered, and no recurrences were observed by follow-up.

When eosinophilic and noneosinophilic effusions were compared, the prevalence of PPI and IPE appeared higher in EPE than in non-EPE. The occurrence of other causes, including PPE and MPE, was similar in the two groups (Table 3).

**Table 3:** Comparison of aetiology of patients with EPE and n-EPE

	Total (n = 252)	EPE (n = 34)	n-EPE (n = 218)	*p*-Value
PPE	36 (14.29%)	3 (8.82%)	33 (15.14%)	0.475
TPE	47 (18.65%)	1 (2.94%)	46 (21.10%)	0.011
MPE	108 (42.86%)	18 (52.94%)	90 (41.28%)	0.201
HR-PE	34 (13.49%)	1 (2.94%)	33 (15.14%)	0.096
CTD-PE	7 (2.78%)	1 (2.94%)	6 (2.75%)	0.951
IPE	6 (2.38%)	5 (14.71%)	1 (0.46%)	< 0.001
PPI	5 (1.98%)	3 (8.82%)	2 (0.92%)	0.014
Others	9 (3.57%)	2 (5.88%)	7 (3.21%)	0.776

Data are presented as n (%)

*EPE* eosinophilic pleural effusion, *n*-*EPE* non-eosinophilic pleural effusion, *PPE* parapneumonic effusion, *TPE* tuberculous pleural effusion, *MPE* malignant pleural effusion, *HR-PE* heart related pleural effusion, *CTD-PE* Connective tissue diseases pleural effusion, *PPI* pleural parasitic infestation, *IPE* idiopathic pleural effusion

### Pleural fluid eosinophilia and malignancy

Comparative analysis of patients with malignant *versus* nonmalignant EPE showed that patients with malignant EPE were significantly older and had a smaller WBC count in the pleural fluid (1.8 vs 4.7 cells × 10^9^/L, *P* < 0.05) (Table 4). However, the percentage of eosinophils in PE was not significantly different between malignant EPE and nonmalignant EPE (*p* = 0.66).

**Table 4:** Comparison of clinical findings of patients with MEPE and non-MEPE, peripheral eosinophilia and normal peripheral eosinophil

	Total (n = 34)	MEPE *vs* non-MEPE			Peripheral eosinophilia *vs* normal peripheral eosinophil		
		MEPE (n = 18)	Non-MEPE (n = 16)	*p*-Value	Peripheral eosinophilia (n = 9)	Normal peripheral eosinophil (n = 25)	*p*-Value
*Age (y)*	56.4 (25–88)	61.7 (44–88)	50.5 (25–83)	0.03	51.7 (25–74)	58.1 (29–88)	0.36
*Sex*				0.774			0.70
Male	20 (58.82%)	11 (61.11%)	9 (77.8%)		6 (66.7%)	14 (56.0%)	
Female	14 (41.18%)	7 (38.89%)	7 (22.2%)		3 (33.3%)	11 (44.0%)	
*Peripheral blood*							
WBC (× 10^9^/L)	8.6 (3.2–21.4)	8.5 (3.2–21.4)	8.8 (4.6–16.7)	0.87	10.1 (4.7–16.7)	8.1 (3.2–21.4)	0.04
LYM (× 10^9^/L)	1.3 (0.3–2.5)	1.4 (0.6–2.5)	1.2 (0.3–2.4)	0.26	1.5 (0.3–2.4)	1.2 (0.5–2.5)	0.13
EO (× 10^6^/L)	514.7 (0–5778.0)	394.4 (0–1500.0)	661.2 (0–5778.0)	0.44	1378.0 (600–5778.0)	204.0 (0–500)	< 0.001
*Pleural effusion*							
LDH (U/L)	369 (90–1402)	449 (172–1402)	280 (90–667)	0.09	437 (128–1126)	345 (90- 1402)	0.51
GLU (mmol/L)	6.07 (3.02–9.77)	6.50 (4.32–9.77)	5.60 (3.02–8.0)	0.12	5.93 (3.02–9.77)	6.12 (3.19–8.34)	0.54
Protein (g/L)	48.0 (27.5–182.9)	55.4 (35.2–182.9)	39.7 (27.5–53.4)	0.07	45.8 (32.8–62.3)	48.8 (27.5–182.9)	0.73
ADA (U/L)	10.4 (0.1–44.7)	8.4 (3.0–29.3)	12.6 (0.1–44.7)	0.24	10.4 (2.8–29.3)	10.4 (0.1–44.7)	0.79
CEA (ng/mL)	106.4 (0.3–2087.0)	199.0 (0.5–2087.0)	2.2 (0.3–4.8)	0.11	24.9 (0.30–162.2)	135.7 (1.0–2087.0)	0.32
WBC (× 10^9^/L)	3.2 (0.1–12.9)	1.8 (0.1–7.67)	4.7 (0.3–12.9)	0.01	5.01 (0.3–12.9)	2.50 (0.1–7.4)	0.10
EO (%)	21.7 (10.0–67.5)	20.6 (10.0–63.0)	22.9 (11.6–67.5)	0.66	31.8 (11.0–67.5)	18.1 (10–46.5)	0.16
RBC (× 10^9^/L)	111.2 (0.2–2986.7)	41.0 (0.2–320)	190.3 (0.2–2986.7)	0.40	27.9 (0.2–216.2)	141.3 (0.2–2986.7)	0.14

Data are presented as the mean (range) or n (%). *MEPE* malignant eosinophilic pleural effusion, *WBC* white blood cell, *LYM* lymphocyte, *EO* eosinophil, *RBC* red blood cell, *LDH* lactate dehydrogenase, *ADA* adenosine deaminase, *GLU* glucose, *CEA* carcinoembryonic antigen

### Pleural fluid eosinophilia and other laboratory parameters

In EPE, there was a positive correlation between the percentage of eosinophils and ADA in PE (r = 0.383 *p* = 0.025), and a reverse correlation between eosinophils and lactate dehydrogenase (LDH) in PE (r = − 0.396, *p* = 0.021). There was a significant correlation between the percentage of eosinophils in peripheral blood and WBC count in PE (r = 0.453, *p* = 0.007). However, there was no correlation between the percentage of eosinophils in PE and peripheral blood (r = 0.29; *p* = 0.09). There were no noteworthy relationships between eosinophils in PE and other laboratory parameters. There was no significant difference between cases of EPE with peripheral eosinophilia and those with normal peripheral eosinophil count (Table 4).

## Discussion

This study included 252 patients with PE diagnosed at a respiratory health research institution, and 34 patients were confirmed to have EPE. To our knowledge, this study represents the largest single study group in our country. Patients with EPE accounted for 13.49% (34/252) of all patients with PE. The percentages were similar to those in previous studies, which reported 12.6% [4] and 10% [2], but higher than those in two other studies, which indicated 5–8% [2] and 7.2% [7]. However, Chu FY found that the morbidity of EPE was only 2.9% [6]. The discrepancy may be attributed to different populations, epidemic characteristics, test methods or the timing of pleural fluid collection. The age of patients with EPE is similar to that in previous reports. The ratio of males to females among EPE patients was 1.43:1, which was lower than the 2:1 ratio reported in previous literature [7]. The higher occurrence of PE in males than in females may explain the ratio of males to females among EPE patients.

EPE can be associated with a wide range of underlying conditions, including infections, malignancies, autoimmune diseases, drug reactions, pulmonary embolism, chest trauma and many others [7]. This study indicates that malignant disease was the leading cause of EPE, followed by PPI, IEPE and PPE in sequence. Compared with non-EPE, the prevalence of IPE and PPI was higher in EPE, while TPE was lower. The aetiological distribution of EPE varies among previous reports. In Krenke’s study, malignancy (34.8%), infections (19.3%), unknown causes (14.1%) and posttrauma (8.9%) were the top four aetiologies in EPE [7]. Oba reported that the most common cause of EPE was malignancy (26%), followed by idiopathic (25%) and parapneumonic (13%) effusions, pleural air/blood (13%), tuberculosis (7%), transudate (7%), other (6%) and CVD (collagen vascular disease) (3%) [5]. Wysenbeek’s study showed that the aetiologies of EPE were trauma (39%), congestive heart failure (14%), infection (8.5%) and idiopathic effusion (8.5%) [10]. The differences in results may be explained by the different prevalences of the aetiology of PE at some institutions [11]. Although there was no drug-induced EPE in our study, the list of drugs associated with EPE include cardiology and internal medicine (warfarin, diltiazem, simvastatin and mesalamine), antibiotics (furantoin, daptomycin and tosufloxacin), psychiatric drugs and neurodrugs (valproic acid, dantrolene) [12–14]. Adverse drug reactions should be considered in the differential diagnosis following thorough investigation for other potential causes of EPE.

The correlation between EPE and malignancy is still a subject of debate. Our study indicated that malignant disease, accounting for 52.94% of EPE cases, was the most common cause associated with EPE. However, it was once believed that the finding of pleural fluid eosinophilia in an exudative effusion considerably reduced the probability of malignancy and conversely increased the likelihood of an underlying benign disorder [15]. Bower and Wysenbeek reported that air/ blood was the most common cause of EPE [10, 15]. However, the spectrum of EPEs has changed since 1960, and malignancy should no longer be considered uncommon among EPEs [5]. The cumulative incidence of malignancy among EPEs has gradually increased from 7 to 25% over the last 4 decades [5]. Current studies (studies performed in the last 2 decades) confirmed that malignancy was the leading aetiology of EPE (accounting for 22.7–40.1% of EPEs) [2, 4, 5, 7, 16, 17]. This tendency may be explained by the development of diagnostic technology, improved diagnostic awareness, disparities in the study populations, or varying disease spectra over time [17].

In a recent study, the majority of malignant pleural effusion (MEPE) was associated with lung cancer. According to a literature review, a vast majority of MEPE is related to solid tumours, and only a small group of patients with haematological malignancies develop EPE [2, 3, 6, 9, 16, 18–21]. Lung cancer and metastatic cancer to the lung were the leading causes of MEPE, including solid tumours from other sites and haematological malignancies. The percentage of MEPE with an unknown primary site of cancer accounted for 5–10% of patients with MEPE [7]. Pathological classification included adenocarcinoma, squamous cell carcinoma, and dysgerminoma [18, 19].

It has also been recognized that a high proportion of idiopathic effusions are characterized by EPE [2, 22]. Ferreiro et al. found that the most frequent aetiology of EPE was known, accounting for 36% of EPE [17]. The percentage of IEPE (14.71%) was just secondary to MPE in a recent study. It has been reported that the prevalence of IEPE varies from 0 to 67% [17, 23]. In the last 2 decades, approximately 3.8–32.1% of EPE cases were diagnosed as IEPE [5, 19, 24]. Thus, as IEPE appeared to be an important part of EPE with obscure pathogenesis, our previous study advocated that complete medical, surgical and drug-related histories should be obtained and thorough work-up and long-term follow-up should be completed to make differential diagnosis [25].

The percentage of PPI (8.82%) was the third largest group of patients with EPE in our study. Three patients had PPI consisting of of patients with infection of *lung fluke Paragonimus westermani, Toxocara spp.*. Consumption of raw or undercooked water or food is the main source of parasite infections. Eosinophilia in PPI has been reported in previous studies of EPEs [26–30], but most of these are case reports or small series reports. Interestingly, in a study of EPEs from mainland China, PPIs were responsible for 31.3% of EPE cases [31]. The high incidence may be explained by the high incidence of patients with PPIs and raw food eating habits in certain areas of the country. Most of the patients with PPI had an exposure history [26, 32]. Therefore, one factor is exposure history. Endemicity and local epidemiology should be taken into consideration when exploring the aetiology of EPE.

This was a retrospective study in a single centre, and the number of patients evaluated was small due to the rare prevalence of EPE. Therefore, a larger, multicentre, prospective study is needed to further explore the epidemic characteristics and clinical significance of EPE.

## Conclusions

In conclusion, malignant disease ranks as the leading cause of EPE, followed by IEPE and PPI. The presence of EPE should not be regarded as a predictive factor of benign conditions. PPIs should be emphasized in areas with a high incidence of parasitic disease.

## Data Availability

The datasets supporting the conclusions of this article are included within the article and tables. Additional data may be available from the corresponding author upon reasonable request.

## Abbreviations

EPE: Eosinophilic pleural effusion
PE: Pleural effusion
n-EPE: Non-eosinophilic pleural effusion
MTB: Mycobacterium tuberculosis
WBC: White blood cell
LYM: Lymphocyte
EO: Eosinophil
RBC: Red blood cell
LDH: Lactate dehydrogenase
ADA: Adenosine deaminase
GLU: Glucose
CEA: Carcinoembryonic antigen
PPI: Pleural parasitic infestation
IPE: Idiopathic pleural effusion
IEPE: Idiopathic eosinophilic pleural effusion
PPE: Parapneumonic effusion
TPE: Tuberculous pleural effusion
HRPE: Heart related pleural effusion
CTD-PE: Connective tissue diseases- pleural effusion
MPE: Malignant pleural effusion
MEPE: Malignant eosinophilic pleural effusion
